# A nonparametric model for quality control of database search results in shotgun proteomics

**DOI:** 10.1186/1471-2105-9-29

**Published:** 2008-01-21

**Authors:** Jiyang Zhang, Jianqi Li, Xin Liu, Hongwei Xie, Yunping Zhu, Fuchu He

**Affiliations:** 1 College of Mechanical & Electronic Engineering and Automatization, National University of Defense Technology, Changsha, 410073, China; 2 State Key Laboratory of Proteomics, Beijing Proteome Research Center, Beijing Institute of Radiation Medicine, Beijing 102206, China

## Abstract

**Background:**

Analysis of complex samples with tandem mass spectrometry (MS/MS) has become routine in proteomic research. However, validation of database search results creates a bottleneck in MS/MS data processing. Recently, methods based on a randomized database have become popular for quality control of database search results. However, a consequent problem is the ignorance of how to combine different database search scores to improve the sensitivity of randomized database methods.

**Results:**

In this paper, a multivariate nonlinear discriminate function (DF) based on the multivariate nonparametric density estimation technique was used to filter out false-positive database search results with a predictable false positive rate (FPR). Application of this method to control datasets of different instruments (LCQ, LTQ, and LTQ/FT) yielded an estimated FPR close to the actual FPR. As expected, the method was more sensitive when more features were used. Furthermore, the new method was shown to be more sensitive than two commonly used methods on 3 complex sample datasets and 3 control datasets.

**Conclusion:**

Using the nonparametric model, a more flexible DF can be obtained, resulting in improved sensitivity and good FPR estimation. This nonparametric statistical technique is a powerful tool for tackling the complexity and diversity of datasets in shotgun proteomics.

## Background

The objective of proteomics is to investigate proteins on a global scale [1,2]. The high-throughput and sensitive tandem mass spectrometry (MS/MS) platform is now a supporting technology for protein identification in proteomic research [3,4]. Using the shotgun strategy, a large number of MS/MS spectra can be gathered in a few hours [5]. The MS/MS data is generally processed by the so-called database searching method [5]. Automated software such as SEQUEST [6] and MASCOT [7] can rapidly assign tryptic peptides to MS/MS spectra by searching a protein sequence database and then identify proteins by utilizing the identified peptides. A notable problem in the MS/MS data processing is the high false positive rate (FPR) of the database search results [8]. Thus, validation of database search results is unavoidable and necessary work, particularly when processing the large amount low accuracy MS/MS spectra with SEQUEST [10].

There are many proposed parameters and algorithms for evaluating SEQUEST database search results [11-32]. Such approaches must confront two main problems: First, the complex physical and chemical mechanisms of the shotgun experiment make it difficult to model the matches between MS/MS spectra and peptides with a one-size-fits-all algorithm [9]. Thus, database search software provides multiple scores, and many empirical and intuitive parameters are used in the validation of database search results. These parameters describe different aspects of the quality of the match and provide complementary information to the validation of the database search results. Combining these parameters while considering their relationships is difficult. Second, many factors can affect the distributions of quality control parameters, including the sample, the database, the experimental conditions, and other random factors [24,27]. Avoiding the effects of such factors during the validation of database search results is difficult. In addition, large-scale proteomics always uses multiple, complementary MS/MS platforms and multiple database search software tools to acquire more results with a high confidence level. Thus, a universal framework for quality control of results is needed [8].

Recently, the randomized database method has become an attractive framework for quality control of MS/MS database search results. By constructing a negative control dataset for each experiment MS/MS dataset and the given database, the randomized database method can provide a universal foundation for the result quality control for different types of database search software and minimize the effects of differences in samples, experiment conditions, and databases [27]. In the randomized database method, the negative control dataset is generated by searching the constructed randomized database and used to simulate random matches from the normal database. The false positive rate can be estimated using the numbers of matches from the normal and randomized database given a set of filter criteria.

Moore et al. used the reverse database (a special kind of randomized database) for their Qscore model in 2002 [20]. Subsequently, Qian et al. [25] and Peng et al. [26] used the reverse database method to investigate the problem of optimizing the cutoff value of *Xcorr *and Δ*Cn *in yeast and human proteome research, respectively. Recently, Higdon et al. [28] investigated some problems encountered in the application of the reshuffled database. As they noted, searching a combined database can yield more accurate FPR estimation than individually searching normal and reshuffled databases. Based on the binomial distribution, Huttlin et al. investigated the minimum error associated with the estimated FPR [33]. They pointed out that the estimated FPR for a large dataset could be quite accurate. Randomized database methods have been widely used in many research projects [34-40]. However, different groups use different criteria; there is no standard statistical framework that can easily integrate commonly used parameters for the quality control of database search results.

There are two primary problems with the randomized database method: how to determine the filter criteria and how to estimate the FPR in succession. Based on the hypothesis that random matches are randomly drawn from normal and randomized databases, formula 1 can be used to estimate the actual FPR [25,26]; Elias et al [27] recommended formula 2 for reliable data quality control:

(1) $$FPR=\frac{N_{R}}{N_{N}}$$

(2) $$FPR=\frac{2N_{R}}{N_{N}+N_{R}}$$

where *N*_*R *_and N_*N *_are the preserved number of peptide matches that pass certain filter criteria and derive from the randomized and normal databases, respectively. Huttlin et al [33] have given a statistical interpretation of formula 2 by using the binomial distribution. So, in this paper, we used formula 2 to estimate FPR. Generally, the filter criteria are discriminant functions (DFs) of database search scores. Determining the acceptance boundaries for database search scores (such as *Xcorr *and Δ*Cn*) is a simple and commonly used method [25,26]. Lopez-Ferrer et al sought to introduce a statistical model that would provide a more complex DF and thus improve the sensitivity of filter criteria [16]. In their model, *XCc*(*=*ln(*Xcorr*)) and $DCc(=\sqrt{\Delta Cn})$ of random matches were considered to follow normal distributions, and the distributions of *XCc *and *DCc *were assumed to be independent. The contour line of the estimated joint distribution of *XCc *and *DCc *was used as the filter boundary. However, we found that normal distributions do not fit well the distributions of *XCc *and *DCc *of the random matches in the LCQ control dataset used in this paper(see "Datasets and database search" section); the *χ*^2 ^goodness of fit test shows that we can reject the null hypothesis *H*_0 _(the distribution is normal) at a significance level of 0.05. Furthermore, the correlation between *XCc *and *DCc *is significant (correlation coefficient = 0.1, p-value = 1.8 × 10^-24^; random matches in the LCQ control dataset, see section "Datasets and database search") which is inconsistent with the independence assumption made by Lopez-Ferrer et al. Another problem with their model is that it cannot be generalized to the situations involving more parameters.

Multivariate nonparametric models can describe data with complex and variable statistical structures. The term nonparametric is not meant to imply that such models do not use any parameters but rather denotes that the number and nature of the parameters are not fixed in advance but flexible. This advantage makes nonparametric models a powerful tool for addressing the problem of multiple parameters with variable distributions in the validation of database search results. Using a set of kernel functions (such as a Gaussian kernel function); the nonparametric model can fit the distribution of multiple parameters directly with considerable accuracy [41,42]. Generally, parameter estimation for a nonparametric model is an iterative optimization procedure. The fully nonparametric probability density function estimate (FnPDFe) procedure proposed by Archambeau et al. [42] and David et al. [43], which is based on a maximum likelihood estimate (MLE) and expectation-maximization (EM) algorithm, is easily implemented with computer programs. In this paper, based on the randomized database searching, FnPDFe was used to estimate the multivariate PDF of the commonly used database scores, the contour lines of the estimated PDF were taken as the candidate DFs. We demonstrated that the FPR estimation errors of the newly introduced method were acceptable on the control datasets from different instruments (LCQ, LTQ and LTQ/FT), its sensitivity was also proved to be improved on the control datasets and the real sample datasets.

## Results

In this section, the DFs of the nonparametric model were discussed at first, and then we show that the sensitivity of the model could be improved by incorporating more features. The accuracy of the FPR estimation of the nonparametric model was investigated and the performance of the nonparametric model was proved superior by comparing with other commonly used methods in proteomics.

### Nonparametric model and the DF

**Figure 1:**
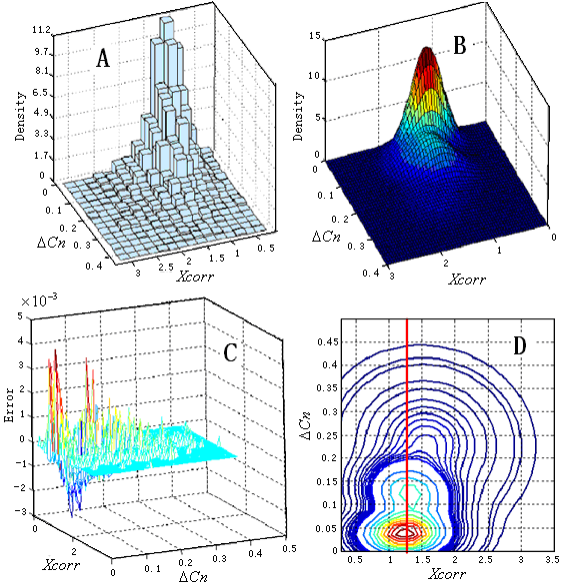
Identified nonparametric model for observations in the control dataset with a +2 charge state. (A) The 2-dimensional histogram. (B) The density function curve of the mixed model with 3 Gaussian functions. (C) The error of the density function in each bin. (D) Contour lines of the density function serve as the filter boundaries.

In order to illustrate the shape of the DFs derived from the nonparametric model, a two dimension model which used *Xcorr *and Δ*Cn *was investigated at first. Because *Xcorr *significantly correlate with the charge state (+1, +2, and +3) [15], the matches with different charge states were processed individually. Since a large percentage of correct matches have a double charge, the matches in the control dataset with a double charge are discussed here. Using a trial and error approach, a model with 3 Gaussian functions (18 variables, Table 1) fit the distribution well (*χ*^2 ^goodness of fit test; significance level = 0.05). Figure 1A and Figure 1B show the histogram and density function, respectively. The estimated error for each bin is shown in Figure 1C. The small error (≤ 3.6 × 10^-3^) also demonstrates that the fit is accurate.

**Table 1:** The model with 3 Gaussian functions for +2 charge observations in the LCQ control dataset

*μ*_*i*_	Σ_*i*_	*P*_*i*_
(1.528008,0.156465)	$\left[\begin{array}{cc} \text{0}\text{.147405} & \text{0}\text{.007248}\\ \text{0}\text{.007248} & \text{0}\text{.004207} \end{array}\right]$	0.138577
(1.615925,0.079976)	$\left[\begin{array}{cc} \text{0}\text{.236614} & \text{-0}\text{.001756}\\ \text{-0}\text{.001756} & \text{0}\text{.001686} \end{array}\right]$	0.476640
(1.369449,0.023879)	$\left[\begin{array}{cc} \text{0}\text{.078369} & \text{-0}\text{.000077}\\ \text{-0}\text{.000077} & \text{0}\text{.000250} \end{array}\right]$	0.384784

DFs that can simultaneously reject as many false positives as possible and accept as many true positives as possible are preferred. Thus, the region in the feature space with fewer random matches is more preferred, and the contour lines of the PDF of the random matches are good candidate DFs (Figure 1D). Generally, random matches have a small Δ*Cn *and *Xcorr*, while correct matches have a large Δ*Cn *and *Xcorr*. Correct matches with the peptide isoform [44] have a small Δ*Cn *and a large *Xcorr*. Matches with a small *Xcorr *and a large Δ*Cn *may be due to the limited search space of the database searching. These matches are rare and more likely to be random matches; they may be localized to the accepted region of the contour line DFs because these results are also rare random events. A new DF of *Xcorr *was added to exclude such matches: *Xcorr *> *m*_*Xcorr*_, where *m*_*Xcorr *_is the mean of *Xcorr *of randomized database matches (bold red vertical line in Figure 1D). Given an expected *FPRα*, a target value *f*_*α *_can be searched to ensure the calculated FPR (*FPR*_*cal*_) is less than or equal to *α*. When searching for *f*_*α*_, *N*_*N *_and *N*_*R *_were counted according to the rules:

(3) $$\sum_{i=1}^{N}P(i)f_{G}(X|i)\leq f_{\alpha }$$

and

**Figure 2:**
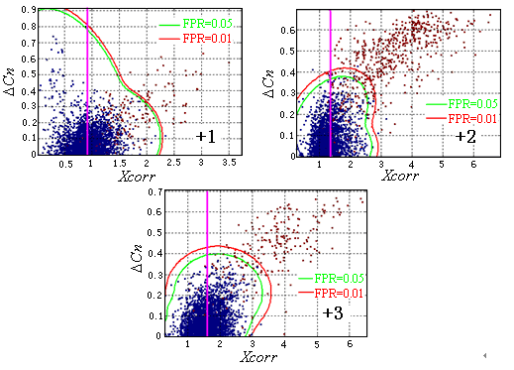
Inferred filter boundaries for different charge state observations in the control dataset. The pink vertical lines in the +1, +2, and +3 panels are the smallest accepted *Xcorr*. The red curves are the filter boundaries for FPR = 0.01, and the green curves are the filter boundaries for FPR = 0.05. The blue points on the *Xcorr*-Δ*Cn *plane represent the randomized database matches, and the red points represent the normal database matches. The shape of the boundaries is greatly different for different charge states.

(4) *Xcorr *> *m*_*Xcorr *_

where *X *= (*Xcorr*, Δ*Cn*) is the observation, and *N *= 3 is the number of Gaussian functions. Many *f*_*α *_satisfied formula 3 and formula 4. The one with the largest *N*_*N *_was used in the final DF. Figure 2 shows the DFs for different expected FPRs and different charge states. The shapes of the boundaries were significantly different, which indicates that it is difficult to fit all the distributions of different charge states with a simple distribution. The nonparametric model can provide feasible solutions to this complex problem. Since the resulting DFs are smooth, this method is more robust than the K nearest neighbor method [41].

### Incorporating more features

One obvious advantage of the nonparametric model is that it can easily integrate more scores for validating peptide identifications. By taking into account more features and performing the classification in a high-dimension feature space, a more reasonable DF can be found, and thus, higher sensitivity can be achieved. Here, another powerful parameter called *Sim *introduced by Zhang [45] in 2004 and discussed by Sun et al. [31] recently was added to the nonparametric model. *Sim *measures the similarity between the experiment and the predicted MS/MS spectrum which was generated by the kinetic model introduced by Zhang [45] and the mass error tolerance for aligning the ions was specified as 0.5.

For the LCQ control dataset, by trial and error, we found a nonparametric model with 5 component GDFs can work well (65 parameters). We also tried a model with 7 component GDFs, but its performance was not improved and two of the component GDFs had a coefficient *P*_*i *_near 0 [see Additional file 1]. Thus, we selected 5 component GDFs to build the model. When the expected FPR was 0.05 and 0.01, the actual FPR was 0.044 and 0.012, respectively. The number of peptide matches after filtering was 765 and 699, which were 104 (approximately 15.6%) and 121 (approximately 20.9%) respectively higher than the results of the nonparametric model using *Xcorr *and Δ*Cn*, respectively. The sensitivity increased to 0.879 and 0.822 respectively, and the specificity did not change. Thus, by incorporating more features, the nonparametric model can provide greater discriminating power. In the following part of this paper, we discussed the nonparametric model with three features: *Xcorr*, Δ*Cn *and *Sim *only. All the model parameters used in this paper were provided in Additional file 1.

### The accuracy of the FPR estimation

The control datasets were generated by analyzing a set of known proteins and peptides with MS/MS platforms, which were commonly used to validate the performance of mathematical models for peptide identification [46]. Table 2 reports the actual FPR and the number of validated matches at two commonly expected FPRs of 0.05 and 0.01. From Table 2, the following propositions can be made:

**Table 2:** Actual FPRs and the corresponding estimated FPRs

Instrument type	Charge state	*Expected FPR = 0.05*			*Expected FPR = 0.01*		
		
		*Total matches/false positive matches*	*Actual FPR*	*Estimated FPR*	*Total matches/false positive matches*	*Actual FPR*	*Estimated FPR*
LCQ	+1	62/3	0.048	0.030	57/2	0.035	0.000
	+2	521/23	0.044	0.049	464/6	0.012	0.009
	+3	181/2	0.011	0.043	178/2	0.011	0.000

LTQ	+1	447/43	0.096	0.048	242/9	0.037	0.008
	+2	4,623/169	0.037	0.050	3,961/26	0.007	0.010
	+3	1,611/59	0.037	0.050	1,449/26	0.018	0.010

LTQ/FT	+1	168/18	0.107	0.047	124/12	0.097	0.000
	+2	1,861/43	0.023	0.049	1,543/14	0.009	0.009
	+3	565/6	0.011	0.048	543/7	0.007	0.007

**Figure 3:**
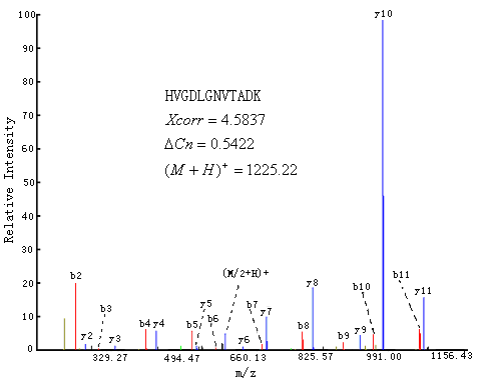
The mass spectrum matched with peptide "HVGDLGNVTADK ".

(1) In most cases, the FPRs estimated by formula 2 were close to but larger than the actual FPRs. Thus, the quality of the resulting datasets was better than claimed. It facilitates the strict result quality control but some sensitivity is lost.

(2) For little datasets, such as +1 charge state matches of different instruments, the actual FPR was larger than the corresponding estimated FPR. The error of the FPR estimation was also a bit larger. This result agrees with the conclusions of Huttlin et al [33].

(3) The estimated FPRs were not equal but close to the expected FPR. The smaller the resulting datasets, the larger the difference between estimated FPR and expected FPR. This arises from the rounding error in formula 2. For example, with an expected FPR of 0.01, the allowable number of random matches was less than 1 for the +1 charge dataset of LCQ, because only 62 matches were left after filtering. Thus, it is impossible to have an estimated FPR exactly equal to 0.01. A preferred alternative is rounding the estimated FPR to 0 (Table 2).

(4) The error of the FPR estimation at the expected FPR of 0.01 is larger that of 0.05. This result means that some unexpected contaminants exist. For example, in the LCQ control dataset, peptide "HVGDLGNVTADK" was identified with high database scores *Xcorr *= 4.5837, Δ*Cn *= 0.542204) and the matched percentage of predicted ions reached 91% (Figure 3). This peptide comes from protein sp|P00441| SODC_HUMAN, which is not a protein in the control sample. But this peptide also belongs to protein sp|P00442|SODC_BOVIN, which may be contaminants in the sample because 4 proteins (ALBU_BOVIN, LACB_BOVIN, LCA_BOVIN and CYC_BOVIN) of bovine were added to the control sample.

(5) Manually checking the confirmed matches by the nonparametric model, we found that some results with large *Xcorr *but very small Δ*Cn *were confirmed. In some cases, the peptide in the second rank was correct. For example, in the LTQ dataset (D2), a peptide "LEAELEK" was identified with *Xcorr *= 2.4273 and Δ*Cn *= 0.0533 (+1 charge state). The peptide at the second rank was "LEALEEK", a peptide from control protein P62937|PPIA_HUMAN, because of the theoretic mass spectrum similarity between these peptides, which will result in some FPR estimation error.

### Compare the performance of nonparametric model with other methods

Two other methods were also be widely used in the proteomic research. The first one (named M1) searches for the optimized cut-off values of *Xcorr *and Δ*Cn *simultaneously while making the number of confirmed matches reached its maximum given an expected FPR. The resulting accepted region on the *Xcorr*-Δ*Cn *plane is a rectangle. The second one (named M2) is Peptideprophet (V1.9), which is an empirical statistic model, introduced by Keller et al [15]. PeptideProphet provided the estimated error rates (EER) at different probability score cut-offs. EER has similar meaning with FPR, so we used it as the measure of the quality of the resulting dataset and only the probability score cut-offs without additional criterion were used to filter the matches. In order to name it easily, we denote the nonparametric model as M3 in the following part of this paper. For the control datasets, the confirmed matches, the actual FPR and the sensitivity were listed in Table 3 (The filter criteria can be found in Additional file 1). Some conclusions can be drawn:

**Table 3:** Comparison of different methods on the control datasets

Instrument type	Methods	*Expected FPR = 0.05*			*Expected FPR = 0.01*		
		
		*Validated matches/false positives*	*Actual FPR*	*Sensitivity *(%)	*Validated matches/false positives*	*Actual FPR*	*Sensitivity *(%)
LCQ	M1	652/30	0.046	74.3	581/15	0.026	69.1
	M2	735/34	0.046	84.1	587/9	0.015	69.3
	M3	765/28	0.037	87.9	699/10	0.014	82.2

LTQ	M1	5507/156	0.028	71.0	4761/48	0.010	62.6
	M2	5818/197	0.034	74.6	4640/20	0.004	61.6
	M3	6681/271	0.041	85.1	5652/61	0.011	74.2

LTQ/FT	M1	2554/69	0.027	83.7	2135/30	0.014	70.9
	M2	2111/46	0.022	69.6	1411/15	0.011	46.8
	M3	2594/67	0.026	87.5	2210/33	0.015	74.5

(1) In each case, the sensitivity of M3 is the highest. The difference in sensitivity of different methods ranges from 3.8% to 27.7%.

(2) For the LCQ and LTQ dataset, the performance of M1 and M2 differs little and Peptideprophet (M2) which was trained by a LCQ control dataset [15] does not seem to work well on the LTQ/FT dataset.

(3) The performance of the nonparametric model differs little on the dataset of different instruments. When the expected FPR is 0.05, the sensitivity is above 0.85 and it is above 0.74 when the expected FPR is 0.01.

(4) FPR estimation errors exist for different methods. In some cases, the error is large. This may be caused by the calculation errors because of unexpected contaminants and random errors.

### Application to large datasets

Shotgun experiments always generate large datasets [8]. Thus, the nonparametric model demonstrated to be effective with the control dataset should be validated using large datasets. At first, we investigated the quality of the confirmed matches by the nonparametric model (The filter criteria can be found in Additional file 7). Another 6 parameters which were commonly used to validate the peptide identifications of SEQUEST database search results were calculated for each match. They are maximal continuous b or y ion series length (*CSL*) [11], the matched percentage of the predicted ions by SEQUEST (*Ions*) [44], ranked preliminary score (*RSp*) [44], the continuity of b or y ion series (*Cont*) [13], the matched percentage of ion intensities in the experiment mass spectrum (*iIons*) [13] and the matched percentage of the peak number in the experiment mass spectrum (*nIons*) [23]. The percentages of the confirmed results which passed the empirical rules (Table 4) convinced us that most of these matches had a high confidence level. It must be noted that *RSp *= 1 is a strict rule [44] and some correct matches may be lost if we require *RSp *= 1. For instance, only 76% correct matches are with *RSp *= 1 in the LTQ control dataset.

**Figure 4:**
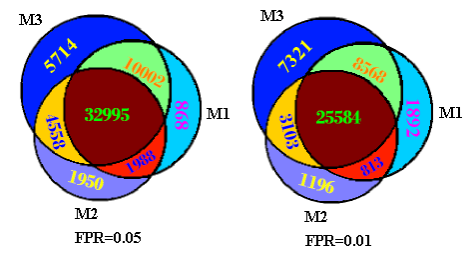
Comparison of the confirmed matches among M1, M2 and M3.

As a case study, we investigated the overlaps of the three methods on the LTQ dataset. More than 90% of the matches confirmed by M1 or M2 were covered by M3 (Figure 4), and 89.1 (FPR = 0.05) and 83.6 (FPR = 0.01) of the matches confirmed by the nonparametric model were covered by M1 ∪ M2. Each method of the three can all provide some matches that are not covered by the other two because they utilize different filter boundaries and different parameters.

**Table 4:** Validate the confirmed matches by empirical rules (%).

	Empirical rules						
Instrument	FPR	*CSL *≥ 4	*Ions *≥ 0.2	*RSp *= 1	*Conts *≥ 0.2	*iIons *≥ 0.25	*nIons *≥ 0.2

LCQ	0.05	92.1	99.5	77.6	86.5	98.0	96.4
	0.01	94.5	99.8	85.9	86.5	98.6	97.6

LTQ	0.05	91.5	90.5	68.6	93.4	89.9	92.7
	0.01	96.9	99.8	75.6	95.6	96.7	97.1

LTQ/FT	0.05	99.1	100.0	67.7	98.6	97.0	99.9
	0.01	99.5	100.0	75.9	99.0	98.0	100.0

**Figure 5:**
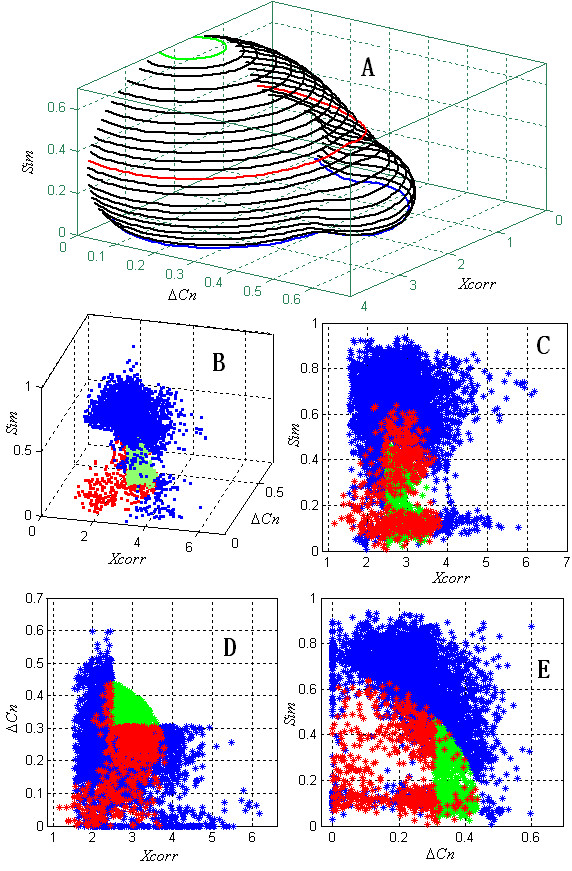
The mesh grids of the DF of M3 and the score distribution of the matches uniquely validated by M1~M3. The blue points in B~E represent the matches uniquely validated by M3, the red points are those of M2 and the green points are those of M1.

Figure 5A shows the mesh grids of a DF of M3 (+2 charge state matches in D5, FPR = 0.01). As it appears, the matches with the smaller *Xcorr*, Δ*Cn *or *Sim *were discarded by M3, which agrees with the experience that the matches with large scores (*Xcorr*, Δ*Cn *or *Sim*) are more possibly correct. Figure 5B~Figure 5E illustrate the score distributions of the matches uniquely confirmed by M1~M3. It is clear that some matches with small *Xcorr*, Δ*Cn *and *Sim *were confirmed by PeptideProphet (red points), which integrated some other parameters, such as preliminary score (*Sp*). M2 confirmed some matches with middle *Xcorr *and Δ*Cn *but small *Sim *(green points). M3 confirmed many matches (4714) with relative smaller *Xcorr *and Δ*Cn *but large *Sim*, which were discarded by M1 and M3. These results demonstrated that different filter boundaries with different parameters would generate different results with different sensitivity and integrating more complementary parameters by appropriate methods could improve the sensitivity of database search result validation.

In Table 5, we gave the numbers of confirmed matches, non-redundant peptides, identified proteins (Minimal protein list assembled by DBParser algorithm [47]) and the percentage of proteins with at least 2 or 3 peptide hits (The filter criteria can be found in Additional file 7). The nonparametric model can confirm up to 14.5% more proteins than the other two kinds of methods, which indicated that our model has a higher sensitivity. For the same kind of instrument, three methods gave about the same percentage of proteins with at least 2 or 3 peptide hits at different confidence levels. The percentage of proteins with at least 2 peptide hits reaches above 50% for the LCQ or LTQ dataset, but it is about 40% for the LTQ/FT dataset. It is interesting that the percentage of proteins with at least 2 or 3 peptide hits can not be improved by improving the confidence level of the peptide identifications when one method is used.

**Table 5:** Comparison of different methods on the complex datasets

Instrument type	Methods	*Expected FPR = 0.05*				*Expected FPR = 0.01*			
		
		*Confirmed matches*	*Non-redundant peptides*	Proteins*	*Proteins with at least 2/3 peptide hits *(%)	*Confirmed matches*	*Non-redundant peptides*	Proteins*	*Proteins with at least 2/3 peptide hits *(%)
LCQ	M1	13,636	5,268	1,922	51.1/35.2	11,512	4,496	1,630	54.0/36.0
	M2	14,128	5,333	1,860	53.7/36.4	10,436	4,219	1,586	53.3/34.4
	M3	15,923	5,872	2,077	52.6/36.2	13,549	5,084	1,729	55.9/38.3

LTQ	M1	45,153	10,359	3,363	54.6/37.6	36,857	8,601	2,733	58.3/39.3
	M2	40,791	10,053	3,166	55.2/39.1	30,696	7,875	2,488	58.7/40.3
	M3	52,569	11,451	3,421	57.9/40.9	44,576	9,756	2,801	61.6/43.1

LTQ/FT	M1	25,672	4,602	2,723	42.0/23.0	22,750	3,869	2,193	42.5/22.8
	M2	23,571	3,947	2,462	45.2/25.4	19,930	3,366	2,083	45.4/24.9
	M3	27,565	4,855	2,820	43.7/24.8	25,185	4,196	2,291	45.6/25.7

Note * It was the count of minimal protein list assembled by DBParser algorithm [47].

## Discussion

Due to the complexity of the peptide identification problem, many parameters have been proposed for use in modeling the quality of matches between MS/MS spectra and peptides. For example, *Xcorr *and *Sim *assess the similarity between theoretical and experimental spectra, and Δ*Cn *assesses the effect of database size. There are two main reasons for the simultaneous existence of multiple parameters. First, the complex physical and chemical process of the MS/MS platform makes it difficult to model the peptide identification problem universally [48]). Second, the huge computational burden of the database search makes it difficult to implement complex models. Thus, most MS/MS data processing approaches currently used include two steps: 1) find candidate peptides quickly and thus reduce the search space; 2) validate the results carefully by taking into account more information. As in this paper, a popular way for quality control of data in shotgun proteomics is to generate a set of easily calculated scores measuring the quality of the matches in different ways and then to combine these parameters to validate the results [23]. The randomized database method provides a feasible framework for constructing a negative control dataset and controlling the FPR of the acquired dataset. The nonparametric model introduced in this paper provides a framework for feature integration and determination of nonlinear DFs. However, if too many parameters are used, the nonparametric model will encounter a computational problem. With too many variable parameters in the model, there may be many solutions to the MLE equations. Thus, the iterative process of the EM algorithm may reach a local minimum, and good performance of the model cannot be guaranteed. Thus, when many features are used, it is recommended that the features be partitioned into different groups by hierarchical clustering [49] and the nonparametric model be applied to each cluster. Other feature-space reduction methods such as principal component analysis (PCA) and partial least squares (PLS) can also be used [50].

**Figure 6:**
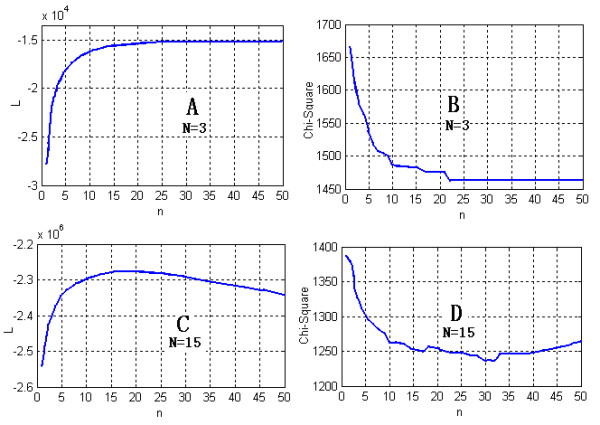
ML function values and *χ*^2 ^statistic with iterative step and different numbers of mixed Gaussian functions. *n*, the iterative step of the EM algorithm; L, the ML function value; chi-square, *χ*^2 ^statistic; N, number of mixed Gaussian functions. It is clear that the EM algorithm will confront the local minimum problem when the number of variables is too many.

The EM algorithm is guaranteed to converge [51]. However, if there are too many variables, it may reach a local minimum. For double-charged matches in the LCQ control dataset (here, we only used two variables: *Xcorr *and Δ*Cn*), we also tried a Gaussian mixed model with 15 components (5 fold of the model we used). The values of the ML function calculated in the iterative process of the EM algorithm increased monotonically for the Gaussian mixed model with 3 components, whereas for the Gaussian mixed model with 15 components they initially increased and then decreased along the iterative step (Figure 6). The performance (*χ*^2 ^statistic; smaller = better) of the 15-mixed models demonstrated the same pattern. It was confirmed that too many variables (90 variables) do not lead to better performance. It is fortunate that the Gaussian model with 3 mixed functions fit the data satisfactorily. For the large dataset and the model with more features, the number of component functions did not exceed 7. If a more complex mixed model is needed, we recommend the following strategies: 1) optimize the ML function directly using more robust nonlinear optimization techniques such as the conjugate gradient and quasi-Newton methods [52]; 2) directly fit the histogram with an optimized binned method (such as Scott's rule [53]) using a RBF neural network; or 3) use another nonparametric model such as the adaptive kernel density estimation proposed by Silverman [54].

The computational burden of the nonparametric model may be doubted, especially for the huge LTQ dataset. It is lucky that it does not need so many observations to build the nonparametric model. If the dataset is too large, we can resample the observations and use fewer observations to build the model. We tried this approach on the LTQ complex dataset. The results achieved by the model built with randomly selected 30,000 observations differed little from that of the model built with all the 432,338 observations. Thus, in the model building procedure, if the number of the observations exceeds 30,000, we resample the dataset and randomly select 30,000 observations to build the model and if the number of the observations is less than 30,000, all the observations are used. Therefore, the consumed time of the model building was less than 2 min on a PC with Intel Pentium 4 2.8G CPU and 512 MB memory.

**Figure 7:**
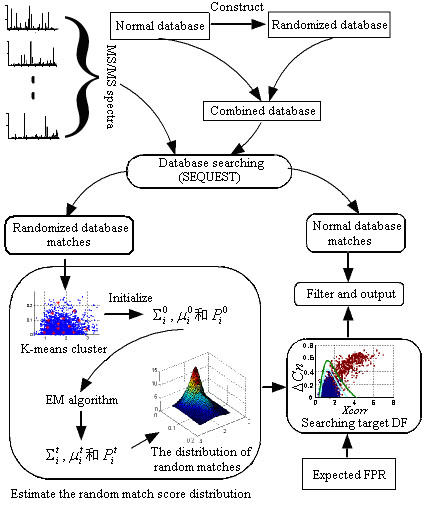
Illustration of the workflow. The workflow is based on the nonparametric model and the randomized database method. First, the randomized database is constructed and merged with the normal database. Then a database search is performed using SEQUEST. Peptide matches from the randomized database are used to build the mixed Gaussian model. Filter boundaries are determined based on the mixed Gaussian model and the expected FPR, and the normal database matches are filtered. During construction of the nonparametric model, k-means clustering is used to initialize the parameters of the EM algorithm. The red points in the left rectangle are the cluster center on the *Xcorr*-Δ*Cn *plane. The red pints on the right rectangle denote the matches from the normal database and the blue points are matches from the randomized database.

The nonparametric model proposed in this paper is easy to use. First, a combined database is prepared containing the normal and randomized protein sequence. Then database search is performed on the combined database and the results are collected; the normal and randomized database matches are labeled with the assistance of references provided by the database search software. The randomized database matches are then used to build the nonparametric model. In this step, a parameter set different from that described here can be used. To obtain the final results, a search for the DF described in the "Nonparametric model and filter boundary" section given an expected FPR is performed. The workflow shown in figure 7 (Methods section) has been implemented by several Matlab (MathWorks, Natick, MA) scripts and in-house C++ programs. The database search results were collected using an in-house program called OutSum.exe, which were stored in the *.out files given by SEQUEST. The resulting data, stored in a plain-text file, were loaded into a Matlab workspace. A script called NoParQ.m was used to build the nonparametric model. The programs used in this paper were provided in a compressed archive [see Additional file 2].

## Conclusion

In this paper, we provide a framework for validation of peptide identification in shotgun proteomics that is based on the randomized database method and a nonparametric model. The practical problems in implementing the nonparametric model were investigated, and its performance was found to be better than that of traditional methods. The nonparametric model can provide a more flexible and accurate solution for DF determination for quality control of large datasets in shotgun proteomics research. All the programs used in this work are available by request from the authors.

## Methods

### Datasets and database search

Six datasets generated by three kinds of mass spectrometry platforms (LCQ, LTQ and LTQ/FT) were used to demonstrate the performance of the nonparametric model. Three control datasets were used to validate the accuracy of the FPR estimation and the improvement of the sensitivity. Since the MS/MS datasets generated by the shotgun technique are always large, we also verified the generality of the nonparametric model on the large real sample datasets. The basic information about the six datasets is listed in Table 6.

**Table 6:** The 6 datasets used in this paper.

*Dataset type*	*Control dataset*			*Real sample dataset*		
Dataset Name	D1	D2	D3	D4	D5	D6
Instrument	LCQ	LTQ	LTQ/FT	LCQ	LTQ	LTQ/FT
Reference or notes	[46]	[55]	unpublished	[44]	[56]	unpublished
Sample	12 purified proteins + 23 peptides	49 purified human proteins	8 purified proteins	Human K562 cell line	Human liver	Human Liver
Data source	the BIATECH Institute (Bothell, WA 98011, USA)	Proteomics Standards Research Group (sPRG) [55]	Beijing proteome Research Center (Beijing 102206, China)	Open Proteomics Database (OPD)[57]	Beijing proteome Research Center (Beijing 102206, China)	Beijing proteome Research Center (Beijing 102206, China)

The two unpublished LTQ/FT datasets were provided by Beijing Proteome Research Center (BPRC). The samples were digested with trypsin and then analyzed by a 7-Tesla LTQ/FT mass spectrometer (Thermo Electron, San Jose, CA) coupled with an Agilent 1100 nano-flow liquid chromatography system. The reverse phase C18 trap columns (300 *μ*m internal diameter × 5 mm long column) were connected with the 6-port column-switching valve for the on-line desalting. A PicoFritTM tip column (BioBasic C18, 5 *μ*m particle size, 75 *μ*m internal diameter × 10 cm long column, 15 *μ*m internal diameter at spray tip, New Objective, Woburn, MA, USA) was used for the following separation. Elution was solvent A (Milli-Q water, 2 % acetonitrile and 0.1%FA, v/v/v) and solvent B (Milli-Q water, 80% acetonitrile and 0.1%FA, v/v/v). The gradient was 15–40% B in 40 min, 40–100% B in 10 min. One FT full MS scan was followed by 5 data-dependent LTQ MS/MS scans on the five most intense ions. The dynamical excluding time was 45 seconds. Ions were accumulated in linear ion trap controlled by AGC. The AGC values were 5 × 10^5 ^charges for FT full MS scan and 1 × 10^4 ^charges for LTQ MS/MS scan. The resolution was 10,000 for FT full MS scan at m/z 400. The temperature of the ion transfer tube was set at 200°C and the spray voltage was 1.8 KV. The isolation width was 4Da and normalized collision energy was 35% for MS/MS scan. Mass spectra were acquired over the m/z range from 400 to 2000.

All the MS/MS spectra were extracted from the *.raw files by Extract_MSn.exe which is a console program in Bioworks 3.2 (Thermo Finnigan, San Jose, CA). For the LCQ datasets, the minimal total ion intensity is 10,000. For the LTQ or LTQ/FT datasets, the total ion intensity of each MS/MS spectrum is required to exceed 100. For all the datasets, the spectra must have at least 20 ions. Then the database search was performed on a local TurboSEQUEST (version 2.7) server. The fixed modification of oxidation (15.99Da) on the Met residue and the variable modification of carboxyamidomethylation (57.02Da) on the Cys residue were set. The enzyme was trypsin and the maximal allowed missed cleavage sites was 2. Only the b and y ions were taken into account. For the LCQ or LTQ datasets, the precursor mass error tolerance was 3.0Da, and for the LFQ/FT datasets, it was 15ppm.

For all the datasets except D2, which was searched against the database published by sPRG [55], the searched databases were derived from IPI Human 3.19 [60]. For the control datasets, the control sequences for dataset D1 and D3 [see Additional file 3, 4 and 5] including the sequences of purified proteins or peptides plus the typical sample contaminants such as keratin and trypsin were added into the IPI Human 3.19. The control sequences for D2 were determined according to the report of sPRG (see Additional file 4) [55]. The databases were constructed using the method proposed in one of our previous paper [58] and could be described as: the protein sequences in the normal database were digested *in silico *(trypsin), and then the amino acid residues (AAR) (except the one on the C-terminal) of the resulting peptides were reshuffled by using a random number generator. Then the reshuffled peptides were spliced to form new protein sequences in the randomized database. Finally, the normal database and the randomized database were merged to form the searched database.

After database searching, the matches with +1, +2 and +3 charge state were extracted (Table 7). For each spectrum, only the first rank match with an assigned peptide with more than 5 AAR was taken into account for further analysis. For the control datasets, the matches which were assigned peptides of control sequences were validated by the following criteria: 1) the b-ion or y-ion series should confirm at least 3 consecutive amino acids of the assigned peptide sequence [12], 2) ranked preliminary score (*RSp*) ≤ 50. The confirmed matches of control datasets were provided in the supplementary materials [see Additional file 6, 7 and 8).

**Table 7:** Database search results of the 6 datasets

*Datasets*		*D1*	*D2*	*D3*	*D4*	*D5*	*D6*
Database search results	+1	467	3,039	1,544	24,875	61,574	36,610
	+2	3,687	28,130	6,028	63,272	754,401	557,994
	+3	3,654	28,943	2,579	63,027	776,794	492,950

### The workflow of the nonparametric model based method

The workflow of the nonparametric model based method is shown in Figure 7. Firstly, a randomized database was constructed by randomizing the tryptic peptide sequence. Then the MS/MS spectra were searched against the combined database using SEQUEST. Then, matches with an assigned peptide from the randomized database (we call them randomized database matches, RDM) were used to build the nonparametric model. The joint distribution of selected parameters (such as *Xcorr*, Δ*Cn *and *Sim *[31,45]) of random matches was fit with the nonparametric model using the FnPDFe method and the contour lines of the estimated PDF, which are complex nonlinear functions, were used as candidate DFs. The actually used DFs were determined according to the expected FPR and formula 2 for different charge states. Finally, the resulting DFs were used to filter the matches from the normal database. In the model-building step, k-means clustering was used to initialize the EM algorithm procedure.

### Initial the nonparametric model with k-means clustering

**Figure 8:**
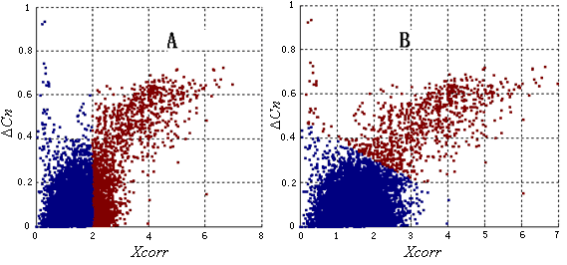
The partitions of k-means clustering before (A) and after (B) normalization (z-score) of the features. Blue and red points represent different clusters. The observations derive from the control dataset. Records with larger *Xcorr *and Δ*Cn *are more likely to be positive results. The partition given by k-means clustering using the observed values is based on *Xcorr*; Δ*Cn *has no effect. After normalization, the partition is more consistent with the empirical knowledge.

K-means clustering [59] is commonly used to partition observations into different groups according to defined distance (such as Euclidean distance). The optimization goal of k-means clustering is to find a partition in which objects within each cluster are as close as possible to each other and as far as possible from objects in other clusters. However, in practice, the scale of each feature will significantly affect the clustering results when Euclidean distance is used. In our application, *Xcorr *and Δ*Cn *were two main features. *Xcorr *is a float point value whose typical value is 2.5 but may be larger than 10; Δ*Cn *is in the range [0, 1]. When directly using the observed values in the k-means clustering, *Xcorr *will dominate the partition results (Figure 8) because the distance (formula 5) between two observations (*Xcorr*_*i*_, Δ*Cn*_*i*_), *i *= 1, 2, is mainly determined by *Xcorr*, which has a larger scale.

(5) $$d=\sqrt{{\left(Xcorr_{1}-Xcorr_{2}\right)}^{2}+{\left(\Delta Cn_{1}-\Delta Cn_{2}\right)}^{2}}$$

Thus, a normalization step, which calculated the z-score of the observed values of each feature, was used to eliminate the scale difference, and thus achieve a more reasonable partition (Figure 8).

### Nonparametric model and the EM algorithm

The basic objective of nonparametric density estimation is to approximate the distribution of observations using the weighted sum of a series of simple functions, which does not emphasize the physical meaning of the parameters but the accuracy of the approximation. This idea can be implemented using smoothing splines or radial basis function (RBF) neural network to fit the histogram directly [41]. Another way to implement the nonparametric model is to fit the distribution with kernel density functions. The optimization goal of the nonparametric model is to minimize the mean integrated squared error of the fit or to maximize the maximum likelihood function of the observations. Many kinds of nonparametric models have been proposed by different researchers [41]. The FnPDFe procedure [42] is attractive because it is easy to implement and has a clear statistical explanation. Let *X *be a *d *dimension random vector *X *∈ *R*^*d*^. Its PDF can be approximated by a Gaussian mixed model that is defined as the linear combination of *N *multivariate Gaussian density functions (MGDFs):

(6) $$f(X)=\sum_{i=1}^{N}P(i)f_{G}(X|i)$$

where:

(7) $$f_{G}(X|i)=\frac{1}{{\left(2\pi \right)}^{d/2}{\left|\Sigma_{i}\right|}^{1/2}}e^{-\frac{1}{2}{\left(X-\mu_{i}\right)}^{T}\Sigma_{i}^{-1}(X-\mu_{i})}$$

and *P*(*i*),*i *= 1,...*N *satisfies: (1) 0 <*P*(*i*) ≤ 1; (2) $\sum_{i=1}^{N}P(i)=1$. *μ*_*i*_, Σ_*i *_is the mean vector and covariance matrix of the *i*-th MGDF.

Consider independent and identically distributed observations set{*x*_1_, *x*_2_,......*x*_*n*_}; the log-likelihood function of the mixed model is:

(8) $$L(\theta )=\sum_{k=1}^{n}\ln f(x_{k})$$

Generally, MLE can be used to infer the parameters *θ *in the mixed model. However, the resulting MLE equations cannot be solved analytically. The FnPDFe method uses the EM algorithm to provide iterative solutions for these parameters [43], which can be read as:

(1) Initial step: Initialize the objective parameters *μ*_*i*_, Σ_*i*_, and *P*(*i*) with heuristic knowledge or random values.

(2) E-step: update the posterior distributions:

(9) $$g^{t+1}(i|x_{k})=\frac{f_{G}^{t}(x_{k}|i)P^{t}(i)}{\sum_{j=1}^{N}f_{G}^{t}(x_{k}|j)P^{t}(j)}$$

(3) M-step: estimate the current parameters:

(10) $$P^{t+1}(i)=\frac{1}{n}\sum_{k=1}^{n}g^{t}(i|x_{k})$$

(11) $$\mu_{i}^{t+1}=\frac{\sum_{k=1}^{n}g^{t}(i|x_{k})x_{k}}{\sum_{k=1}^{n}g^{t}(i|x_{k})}$$

(12) $$\Sigma_{i}^{t+1}=\frac{\sum_{k=1}^{n}g^{t}(i|x_{k}){\left(x_{k}-\mu_{i}^{t}\right)}^{T}(x_{k}-\mu_{i}^{t})}{\sum_{k=1}^{n}g^{t}(i|x_{k})}$$

**(4) **Repeat steps 2–3 until the change of parameters is very little.

One problem with implementation of the EM algorithm is how to initialize the parameters. Use of an improper starting point may prolong the converging time of the EM algorithm or cause it to reach a local minimum. In this work, k-means clustering was used to partition the observations into subclasses, and the means and covariance matrixes of the component Gaussian distributions were initialized using the means and covariance matrixes of the subclasses.

Another difficulty in implementing the EM algorithm is the selection of the number of component density functions. Generally speaking, inclusion of more functions will approximate the distributions of the observations more accurately, while allowing more parameters to be determined. However, overly complex models may cause the EM algorithm to reach a local minimum and worsen the performance of the resulting model. In this work, a trial and error procedure was used to select the minimum number of component density functions: try numbers from 2 until the change of the likelihood function value is very little (such as less than 1%).

## Abbreviations

MS/MS: tandem mass spectrometry; DF: discriminate function; FPR: false positive rate; LCQ: 3D quadrupole ion trap; LTQ: linear trap quadrupole; FT: Fourier transform; PDF: probability density function; FnPDFe: fully nonparametric probability density function estimate; MLE: maximum likelihood estimate; EM: expectation-maximization; MGDF: multivariate Gaussian density function; RDM: randomized database matches; IPI: international protein index; MLE: maximum likelihood estimate; EM: expectation-maximization.

## Authors' contributions

JZ developed the program for data processing and wrote the main text of the paper. XL finished the experiment to analyze the samples on LTQ/FT platform. HX inspected all the algorithm problems and provided abundant suggestions for improving the implementation of the EM algorithm. YZ and FH reviewed the paper and revised its framework.

## Supplementary Material

Additional file 1 The parameters of the nonparametric models for different datasets. This file collected the parameters of the nonparametric models and filter criteria for different datasets. The file was compressed as RAR archive to reduce the size. Click here for file

Additional file 2 Program package. This file packaged all the programs used in this work, which include the Microsoft Windows executable EXE files and the Matlab script M files. A readme file is provided in this package to illustrate how to use these programs. Click here for file

Additional file 3 The control sequences of the LCQ control dataset. This file includes the control sequences for the LCQ control dataset, which include the sequences of control proteins and the common contaminants. The file was compressed as RAR archive to reduce the size. Click here for file

Additional file 4 The control sequences of the LTQ control dataset. This file includes the control sequences for the LTQ control dataset, which include the sequences of control proteins and the common contaminants. The file was compressed as RAR archive to reduce the size. Click here for file

Additional file 5 The control sequences of the LTQ/FT control dataset. This file includes the control sequences for the LTQ/FT control dataset, which include the sequences of control proteins and the common contaminants. Click here for file

Additional file 6 Validated matches in the LCQ control dataset. This file contains the validated correct matches for the LCQ control dataset. The file was compressed as RAR archive to reduce the size. Click here for file

Additional file 7 Validated matches in the LTQ control dataset. This file contains the validated correct matches for the LTQ control dataset. The file was compressed as RAR archive to reduce the size. Click here for file

Additional file 8 Validated matches in the LTQ/FT control dataset. This file contains the validated correct matches for the LTQ/FT control dataset. The file was compressed as RAR archive to reduce the size. Click here for file
